# Bladder cancer precision-cut tumor slices (PCTS) preserve functional immune microenvironment enabling ex vivo drug evaluation

**DOI:** 10.1186/s12916-026-05173-4

**Published:** 2026-08-29

**Authors:** Sarah Richtmann, Maja Radak, Viktor Sincic, Jenny Hugoson, Nadja Werner, Valeriia Skoryk, Darcy Wagner, Fredrik Liedberg, Kristina Lundberg

**Affiliations:** 1 Department of Immunotechnology, Lund University, Lund, Sweden; 2 CREATE Health Cancer Center, Lund University, Lund, Sweden; 3 LUCC Lund University Cancer Center, Lund University, Lund, Sweden; 4 Department of Experimental Medical Sciences, Lund University, Lund, Sweden; 5 Department of Biomedical Engineering, McGill University, Montreal, Canada; 6 Department of Translational Medicine, Lund University, Malmö, Sweden; 7 Department of Urology, Skåne University Hospital, Malmö, Sweden

**Keywords:** Ex vivo models, Bladder cancer, Drug development, Tumor microenvironment, Immunotherapy, Precision-cut tumor slices

## Abstract

**Background:**

The development of effective cancer immunotherapies is hindered by the lack of preclinical models that faithfully preserve tumor-immune interactions. Current approaches provide limited representation of immune components, creating a critical translational gap between laboratory findings and clinical outcomes. Precision Cut Tumor Slices (PCTS) offer a promising solution by maintaining complete tumor architecture including native immune cell populations and spatial organization.

**Methods:**

We established a PCTS platform using fresh bladder cancer specimens and optimized culture conditions to preserve tissue integrity and cell viability. Comprehensive phenotyping was performed using flow cytometry to characterize tumor and immune cell populations. Immune functionality was validated through CD3/CD28 stimulation experiments measuring activation markers (CD69, CD137, HLA-DR) and cytokine secretion. Nine patient samples were treated with the PD-1 checkpoint inhibitor nivolumab to assess ex vivo effects. Additionally, multiplex immunofluorescence microscopy was employed on fixed PCTS as a readout for spatial distribution and cytotoxic effects of chemotherapy agents.

**Results:**

PCTS maintained viability of both cancer cells and diverse immune populations for up to six days. Functional validation demonstrated immune responsiveness upon T cell stimulation, with elevated activation markers and significantly increased secretion of inflammatory mediators including IFNγ and CXCL10, in treated as compared to control samples. The platform successfully addressed intra-tumor heterogeneity while maintaining experimental reproducibility across patient samples. Treatment with nivolumab revealed heterogeneous responses, with two out of nine samples (22%) demonstrating dose-dependent immune activation in the PCTS model, consistent with published response rates. Multiplex immunofluorescence analysis confirmed the ability to capture both immune-mediated responses and direct cytotoxic effects on cancer cells, enabling comprehensive evaluation of combination therapies.

**Conclusions:**

This validated PCTS platform represents a versatile human-derived tool for evaluating cancer immunotherapies while maintaining physiological tumor microenvironment complexity. The technical foundation established here enables future prospective studies correlating ex vivo responses with patient clinical outcomes, potentially advancing personalized treatment selection and reducing reliance on animal models in immunotherapy development.

**Supplementary Information:**

The online version contains supplementary material available at https://doi.org/10.1186/s12916-026-05173-4.

## Background

The development of effective anti-cancer therapies remains hindered by the significant translational gap between preclinical research and clinical outcomes, particularly for immunotherapeutic agents that require complex interactions within the tumor microenvironment (TME). Current preclinical models present substantial limitations: conventional cell lines lack the spatial organization and immune contexture essential for evaluating immunotherapies, while tumor organoids, despite preserving some architectural features, also lack interacting immune context or provide only limited representation of immune cell populations [1, 2]. Animal models, though incorporating immune responses, suffer from species-specific differences that often fail to predict human therapeutic responses, particularly for immunomodulatory agents targeting human-specific pathways [3, 4].

Bladder cancer represents a significant global health burden with projected further increasing incidence [5]. Despite therapeutic advances, the disease continues to pose substantial clinical challenges, with high mortality rates in muscle-invasive bladder cancer (MIBC) and local recurrence rates up to 70% after bladder-sparing treatment in non-muscle-invasive bladder cancer (NMIBC) [6]. The introduction of immune checkpoint inhibitors (ICIs) targeting PD-1 and PD-L1has revolutionized treatment paradigms for advanced disease both in the metastatic and curative setting [7, 8]. However, response rates to approved ICIs remain at approximately 20% in bladder cancer patients [9, 10], highlighting the urgent need for improved patient stratification and novel therapeutic approaches. Combining ICI with cancer-targeted therapy has demonstrated enhanced efficacy [11], underscoring the need for translational models to evaluate immunomodulatory treatments both alone and in combination with other therapies.

Preserving the TME is crucial for bladder cancer models as it is a critical determinant of both disease progression and treatment response. Bladder tumors exhibit complex spatial organization with heterogeneous distribution of immune cells, stromal components, and varying levels of immune checkpoint expression across different molecular bladder cancer subtypes [12, 13, 14]. The intricate interplay between tumor cells and their surrounding microenvironment, particularly immune cell populations, influences prognosis and treatment outcomes [15, 16]. Accordingly, preclinical models that adequately capture the complete human bladder TME are pivotal for accurate evaluation of novel immunotherapeutic strategies. By maintaining the three-dimensional architecture of the tumor alongside its original microenvironment, including immune cells, stromal components, and extracellular matrix, Precision-cut tumor slices (PCTS) provide a physiologically relevant platform for studying complex tumor-immune interactions, with high predictive accuracy for treatment response in different settings [17, 18].

We establish and validate an ex vivo and patient-derived bladder cancer model using PCTS. Additionally, we explore different outcome measures for response to T cell stimulation, therapeutic antibodies including ICIs and chemotherapy. The PCTS bladder cancer model maintains the original cellular composition, tissue architecture, and functional immune compartments including preservation of viability and functionality of tumor-infiltrating immune cells, allowing for a detailed analysis of immune responses. Thus, the model provides a useful tool for evaluating complex cell responses to immunogenic agents, chemotherapy, and novel treatment combinations, potentially bridging the translational gap between preclinical findings and clinical outcomes in bladder cancer immunotherapy.

## Methods

### Patient characteristics and molecular subtype classification

Tumor samples from 23 patients with primary bladder cancer were obtained by cold-cup biopsies from the exophytic tumor portion between 2023 and 2024 at Skåne University Hospital, Malmö, and Landskrona Hospital, Landskrona, Sweden. Tumors were classified according to the Lund Taxonomy based on IHC and/or bulk RNA sequencing within the UROSCANSEQ-infrastructure [19].

The median age was 76 (Inter Quartile Range 72–78) years, five (22%) were female, and all but four tumors were 30 mm or larger in diameter. Two patients were treated for bacteriuria within two weeks of sampling. Stage and grade distribution is given in Table 1. Subsequently, 10 patients (43%) were treated with radical cystectomy, and the remaining with a bladder sparing approach.

**Table 1:** Patient cohort data

Sample ID	Sex	Age	Stage	Grade (WHO 1999)	cN	M	Molecular subtype Lund Taxonomy
P1	M	67	T1 + Cis	G3	N0	M0	UroA
P2	M	63	Ta	G2	N0	M0	UroA
P3	F	76	T3 + Cis	G3	N2	M0	UroA
P4	M	78	T1	G2	N0	M0	UroA
P5	M	84	T1	G3	N0	Mx	UroA
P6	M	73	T1	G3	N0	M0	UroB
P7	M	59	T3 + Cis	G3	N1	M0	n/a
P8	M	77	T1	G3	N0	M0	BaSq
P9	F	66	T3	G3	N0	M0	UroA
P10	M	73	T2 + Cis	G3	N0	M0	n/a
P11	M	77	T1 + Cis	G3	N0	M0	n/a
P12	M	78	Ta	G2	N0	Mx	n/a
P13	M	74	T1 + Cis	G3	N0	M0	n/a
P14	M	71	Ta	G2	N0	Mx	UroA
P15	M	84	T2 + Cis	G3	N0	M0	n/a
P16	M	69	T3 + Cis	G3	N0	M0	BaSq
P17	F	76	Ta	G2	N0	Mx	UroB
P18	M	81	T2	G3	N0	M0	BaSq
P19	M	77	Ta	G2	N0	M0	n/a
P20	M	75	T1 + Cis	G3	N0	M0	n/a
P21	F	77	T2	G3	N0	M0	GU
P22	F	78	T1 + Cis	G3	N0	M0	UroA
P23	M	79	T4a + Cis	G3	N0	M0	UroB

Cis = Carcinoma in situ; UroA = Urothelial-like A; UroB = Urothelial-like B, BaSq = Basal/Squamous; n/a = not available

### Generation and culturing of PCTS

After sampling, tumors were immediately placed in MACS Tissue Storage Solution (Miltenyi Biotec, Bergisch-Gladbach, Germany) and transported to the laboratory on ice. Prior to slicing, tumors were embedded in a 3% low gelling temperature agarose cylinder (Sigma Aldrich, St. Louis, Missouri, United States) in D-PBS w/o Ca, Mg (Cytiva, Marlborough, Massachusetts, United States). Tumors were cut into 300 μm slices in 1 x DPBS on ice using a vibratome (Model 7000smz-2, Campden Instruments, Loughborough, England) (70 Hz frequency, 1.50 mm amplitude, 0.19 mm/s advance).

**Fig. 1:**
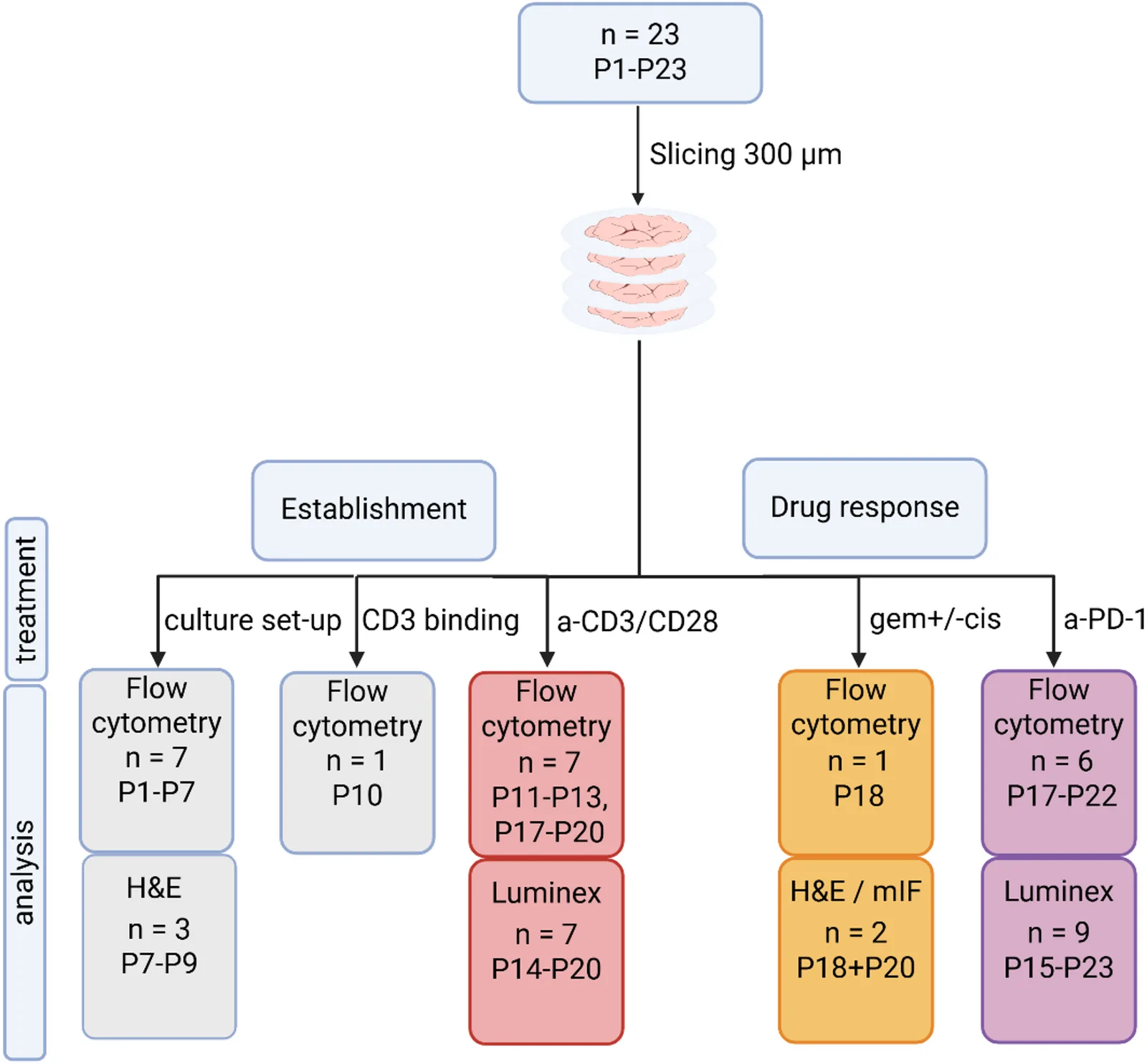
Schematic representation of the PCTS workflow. Primary bladder cancer samples were obtained by cold-cup biopsy and embedded in low-gelling agarose. Using a vibratome, precision cut slices of 300 μm thickness were generated and subjected to one of the indicated experimental arms. Analyses were performed using hematoxylin and eosin (H&E) staining in formalin fixed paraffin embedded (FFPE) samples, flow cytometry of dissociated tissue, and measurement of secreted cytokines and chemokines in the slice culture supernatant (Luminex)

Slices were randomly selected in duplicates or triplicates and put on Millicell cell culture inserts with 0.4 μm pore size (Sigma Aldrich) in 6-well cell culture plates (Costar, Corning Incorporated, New York, United States) containing 2 mL of media in the bottom and 1 mL in the insert and kept at 37 °C and 5% CO_2_ for one hour recovery. Media was exchanged in the well after one hour and again after overnight culture. A complete overview of tissue processing can be found in Fig. 1.

### Preservation and media composition

To select a suitable medium for ex vivo culturing of the bladder slices, four different media were tested in nine tumor samples, and the preservation was evaluated for a period of six days. Samples were compared to a reference sample from day zero. Media was exchanged on day one and three. On day three and six, slices were collected and further processed to formalin fixed paraffin embedded (FFPE) blocks and/or for flow cytometry evaluation.

### Generation of FFPE tumor slices and H&E staining

After ex vivo culturing, bladder slices were formalin-fixed and embedded in paraffin. The slices were thus folded in tissue wrap and fixed in formalin for 24 h. The FFPE blocks were sectioned with a thickness of 4 μm using a microtome and Hematoxylin & Eosin stained by HistoHub, Division of Oncology, Department of Clinical Sciences Lund, Lund University. H&E slides were imaged in bright field using an Olympus APX100 Microscope (Olympus, Hamburg, Germany) at 20x magnification.

### Histopathological evaluation of PCTS

We established a scoring system based on four key histopathological criteria: (I) overall tissue and cellular preservation compared to the initial condition – assessing how closely histological structure resembles the original sample; (II) distinguishable tumor pattern(s) – evaluating whether characteristic histological features remain recognizable; (III) preservation of pattern distribution – indicating how spatial distribution of histological structures is maintained; and (IV) necrosis progression – tracking necrosis extension at cellular and tissue level. Scoring was performed across all available fields of view per sample.

### Flow cytometry

Bladder tumor slices were dissociated to obtain a single cell suspension, and apart from samples P1-3 and P7-8 which were analyzed fresh, all samples were cryo-preserved before analysis. Briefly, slices were cut in RPMI media (Cytiva) (supplemented with 0.1 mg/mL gentamycin) with scissors to produce 0.5 × 0.5 mm tissue pieces. Further dissociation was performed with DNase I (200 Kunitz/mL) (Sigma Aldrich) and collagenase IV (2 mg/mL) (Sigma Aldrich) for 20 min at 37 °C and 5% CO_2_. Then, the cell suspension was filtered through a 70 μm cell strainer, centrifuged, resuspended in cryomedia (70% RPMI with gentamycin, 20% FBS and 10% DMSO) and placed in the vapor phase of liquid nitrogen for 24 h before being moved to liquid nitrogen until analyzed.

Cryopreserved samples were thawed and incubated at 37 °C and 5% CO_2_ in MACS buffer (PBS, 0.5% BSA and 250 µM EDTA) for 10 min prior to staining. Fresh samples (P1-3 and P7-8) were processed and stained directly after dissociation using the same staining workflow. The single cell suspensions were stained with a viability dye, blocked with mouse IgG (Jackson ImmunoResearch, West Grove, Pennsylvania, United States) and further stained with a 12-plex or 22-plex antibody panel (Additional file 1: Table S1 and Table S2**).** Cells were stained for annexin V in an annexin V Binding Buffer (Biolegend, San Diego, California, United States) in a last separate step. All washing steps were performed with MACS buffer. Afterwards, the cells were analyzed using a flow cytometer (BD FACS Aria II or Cytek Aurora).

### Treatment with immune stimulatory agents, checkpoint inhibitor, and chemotherapy

After overnight recovery, slices were treated with anti-CD3 and anti-CD28, nivolumab, cisplatin, gemcitabine or cisplatin + gemcitabine (Additional file 1: Table S3) in fresh media for 24 h at 37 °C and 5% CO_2_. After treatment, slices were further processed, e.g. for flow cytometry and/or FFPE, and culture supernatants were collected for analysis by multiplex immunoassay.

### Multiplex immunoassay (Luminex)

To guide selection of analytes for focused analysis, an initial screening was performed using a broader 46-plex Luminex panel on a subset of PCTS samples (P14-17, P21, P22). Analytes that were detectable across samples were selected for subsequent analysis using a focused 26-plex panel. Analytes that were consistently outside the assay detection range were not included in the focused panel, and as reliable values could not be obtained for these analytes, they were excluded from downstream analysis and are therefore not reported.

Supernatants were collected from each well, centrifuged at 300 x g for 5 min, transferred to a fresh tube, and frozen at − 20 °C for batch analysis by multiplex immunoassay. Concentrations of cytokines and chemokines were analyzed in duplicates using the Luminex Human XL Cytokine 46-plex Fixed Panel (Bio-techne R&D Systems, Catalog # LKTM014B, Minneapolis, MN, United States), or customized Luminex Human Premixed Multi-Analyte Kit 26-plex (Bio-techne R&D Systems) according to the manufacturer’s protocol in a Bio-plex 200 system (Bio-Rad, Hercules, California, USA). Analytes included in the customized Luminex Human Premixed Multi-Analyte Kit 26-plex were MCP-1, MIP-1β, MIP-3α, GROβ, Flt3L, GM-CSF, IFN-α, IL-1α, IL-1ra, IL-6, TNF-β, PDGF-AA, TNF-α, MIP-1α, MIP-3β, GROα, IP-10, G-CSF, Granzyme B, IFN-γ, IL-1β, IL-2, IL-10, PD-L1, PDGF-BB and VEGF-A.

### Immunofluorescence microscopy and image analysis

To assess T cell infiltration patterns and cellular responses (proliferation and apoptosis) in bladder cancer PCTS following different treatments, we utilized a validated 5-plex immunofluorescence antibody panel (Additional file 1: Table S4). All antibodies underwent validation and titration procedures using FFPE bladder tumor specimens prior to implementation.

For tissue processing, FFPE sections underwent standard deparaffinization using Histolab clear (Histolab, Puente St, Brea, CA, USA) followed by sequential rehydration through graded ethanol solutions (100%, 95%) and deionized water. Heat-induced epitope retrieval was performed using 1x IHC Antigen Retrieval Solution (10x high pH, Thermo Fisher) in a TintoRetriever pressure cooker (Bio SB, Santa Barbara, California, USA) at high temperature and pressure settings (114–121 °C) for 15 min. Following antigen retrieval, sections were rinsed with 1x TBS-T (Tris Buffered saline with Tween 20, Cell Signaling Technology, Danvers, MA, USA) and blocked with TBS-T containing 1% BSA to prevent non-specific binding.

Primary antibody incubation was conducted overnight at 4 °C using the 5-plex panel. Post-incubation washing with 1x TBS-T preceded nuclear counterstaining with DAPI (Thermo Fisher). Slides were coverslipped using Fluoromount-G Anti-Fade Mounting Medium (SouthernBiotech, Birmingham, AL, USA) and allowed to dry overnight before imaging.

Fluorescence imaging was performed using an Olympus APX100 Microscope (Olympus) at 20x magnification. Multichannel acquisition covered regions of interest encompassing the entire tissue section, with 5–10 manual focus reference points established to ensure optimal image resolution throughout the scanning area.

Image analysis employed InstanSeg cell segmentation software (QuPath extension) utilizing a pre-trained fluorescence model [20] that processed all five channels simultaneously: 395 nm (DAPI), 488 nm (panCK), 592 nm (Ki67), 650 nm (Cleaved Caspase-3), and 740 nm (CD3). Cell classification was achieved through QuPath-based machine learning classifiers trained individually for each marker using 50–100 representative positive and negative cells selected from regions with diverse cellular densities, background conditions, and signal intensities within the PCTS specimens. For supplementary multiplex immunofluorescence analysis of sample P20, tissue sections were imaged using an Olympus VS200 microscope with a 20x objective. One PCTS per treatment was included for analysis. Image analysis employed InstanSeg cell segmentation software, utilising a pre-trained fluorescence model that processed three channels (DAPI, panCK, CD3). Cell classification was achieved through machine-learning classifiers trained individually for each marker using 50–200 representative positive and negative cells.

### Data analysis and statistical test

Histological scores for each tested PCTS condition were analyzed using a Python script in Jupyter Notebook: data normalization, calculation of median values with interquartile range (IQR) and pairwise comparisons between media at Day 3 and Day 6 by Mann-Whitney U Test.

Flow cytometry data was analyzed using FlowJo software (Becton, Dickinson & Company, version 10.10.0). Samples with viability below 5% and high autofluorescence resulting in unreliable gating were excluded. Representative gating strategy for the 12-plex panel is depicted in Additional file 1: Figure S1. CD3/CD28-treated samples were analyzed with an adjusted gating strategy shown in Additional file 1: Figure S2.

Data was analyzed using GraphPad Prism (version 10.6.0). The concentrations for each analyte obtained by the multiplex immunoassay were normalized by setting the highest value for each analyte to 1 and the lowest value to 0. Values with coefficient of variation (%CV) in duplicate measurements above 20% were excluded from the analysis. Correlation between paired culture wells was assessed using Spearman rank correlation (non-parametric approach as analyte concentrations do not follow a Gaussian distribution, and data points represented paired measurements from individual patient-derived cultures rather than independent samples).

The differences in percentage of viable cells, cancer cells, immune cells, T cells and myeloid antigen-presenting cells (APCs) were evaluated using a Friedman’s (non-parametric) test with multiple comparison correction by a Two-Stage set-up method of Benjamini, Krieger and Yekutieli. Adjusted *P* values < 0.05 were considered statistically significant.

## Results

### Validating PCTS cellular viability and tissue integrity

**Fig. 2:**
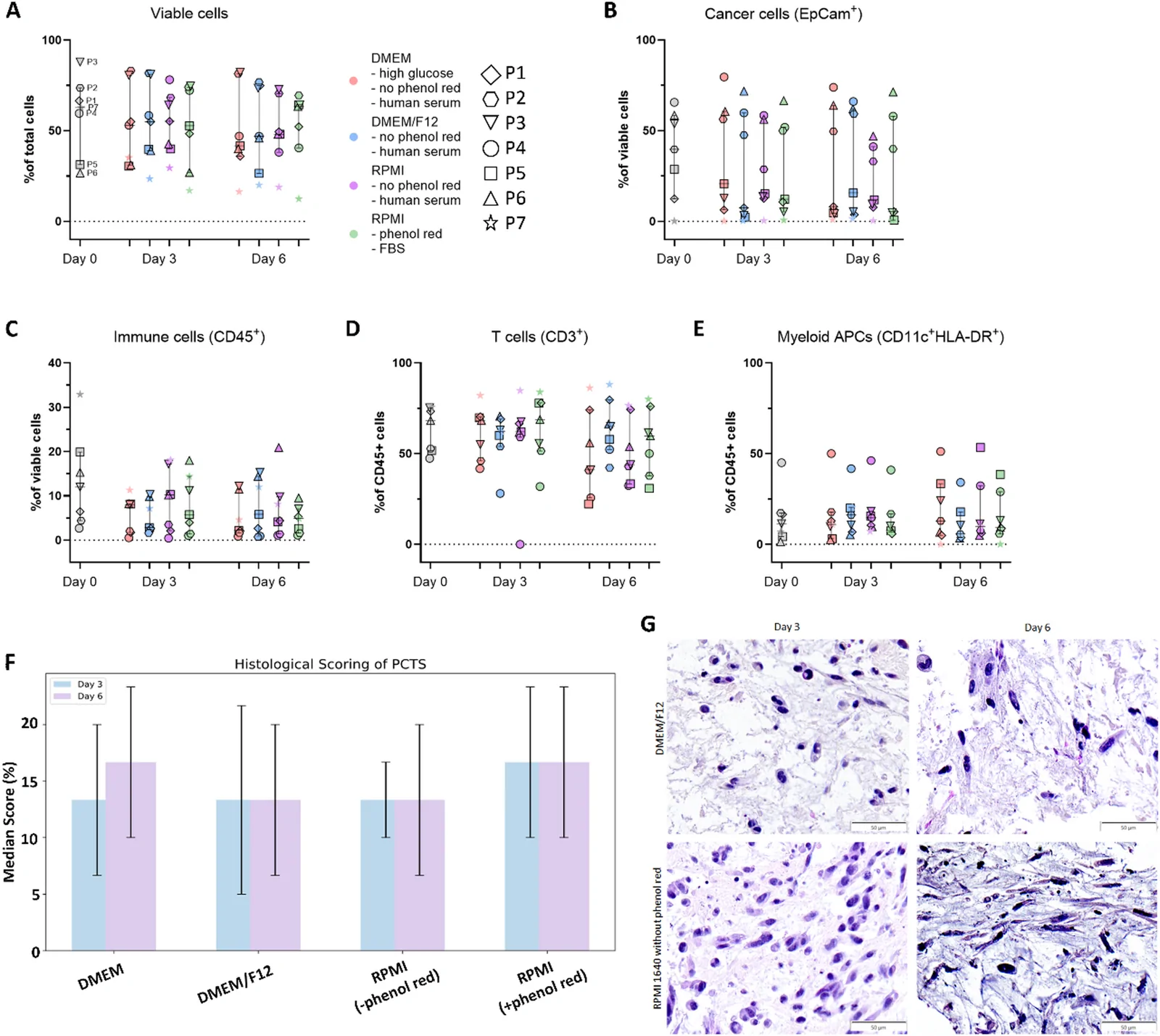
Establishment of culture conditions for PCTS.** A**-**E** Cell viability was assessed with a viability dye and measurement of viable cells on a flow cytometer. The graphs depict total (**A**) viable cells, (**B**) viable cancer cells, (**C**) immune cells, (**D**) T cells, and (**E**) myeloid cells, on day zero compared to three and six days of cultivation. Colors represent the different culture media tested. Each shape corresponds to a patient derived slice culture (P). P1-3 and P7 were analyzed freshly and not cryo-preserved. (**F**) Histological comparison of media performance for preserving PCTS on day three and day six. Bars represent the median total score based on four histological semi-quantitative criteria. Higher scores reflect decreased morphological preservation. (**G**) Representative H&E-stained PCTS sections after three and six days in culture in DMEM/F12 and RPMI 1640 without phenol red, respectively. Scale bar corresponds to 50 μm

To establish a suitable culture protocol ensuring cell viability and maintenance of tissue integrity, we first tested four different culture media (Additional file 1: Table S5) for up to six days. Using a viability dye and flow cytometry, we assessed effects on PCTS that were dissociated after culture. When analyzing the specific samples, each depicted by a shape in the graphs, and following the data from baseline over time and in the different culture media, the initial cell viability within the PCTS sample was not significantly influenced by the culture protocol irrespective of the tested media and time points (Fig. 2A). Evaluation of distinct cell populations revealed that both EpCam+ cancer cells and CD45 + immune cells present in the PCTS were viable in the different conditions (Fig. 2B and C). Importantly, the relative proportion of both T cells and myeloid cells remained at baseline levels after three and six days (Fig. 2D and E). Comparison of fresh and cryo-preserved samples revealed no systematic reduction in post-thaw viability (Additional file 1: Figure S3).

To evaluate the extent of spatial tissue preservation, histological features were scored by a pathologist in H&E stainings of formalin-fixed PCTS sections after culture (Fig. 2F). Overall, a trend of progressing necrosis was observed in the examined PCTS at different time points, characterized by an increase in shadow cells and features of cytoplasmic and extracellular coagulation, alongside a reduction in recognizable histological patterns. Additionally, a gradual decrease in sample size was noted over six days. Among the four investigated culture media, PCTS cultured in DMEM/F12 and RPMI 1640 without phenol red showed the best tissue preservation, maintaining tissue architecture and cellular integrity for up to six days (Fig. 2G). However, the established scoring system did not result in statistically significant differences in median scores between the tested conditions. Notably, one patient’s PCTS in DMEM/F12 exhibited a well-preserved tissue focus despite initial prominent necrotic areas (Additional file 1: Figure S4).

Based on these findings, we conducted the following experiments in a mixed culture medium (DMEM/F12 + RPMI 1640, ratio 1:1). The initial cell viability was preserved throughout the culture period, with no observable impact of the culture protocol. For downstream functional analyses, we applied a standardized short-term format consisting of overnight recovery followed by a 24 h treatment/stimulation period. This time window was selected to minimize confounding effects of progressive necrosis observed during longer culture, while allowing sufficient time for antibody penetration and detection of early immune activation readouts.

### Overcoming intra-tumor cell composition heterogeneity

**Fig. 3:**
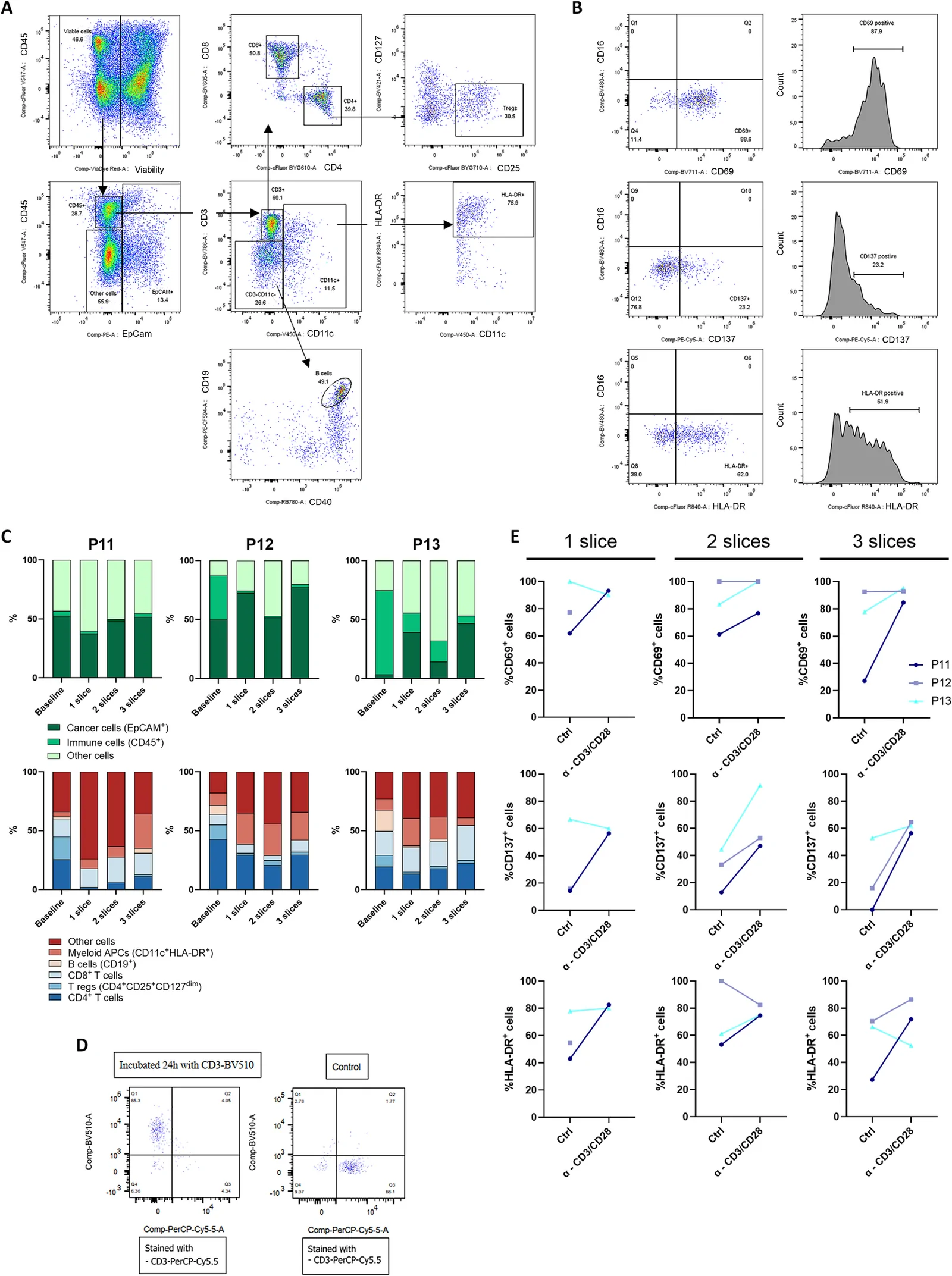
Assessment of assay robustness using high-plex flow cytometry. **A** Representative gating strategy of a sample stained with a 22-plex antibody panel and analyzed using flow cytometry. **B** Dot plots and corresponding histograms of three surface activation markers on CD3 + T cells. **C** Stacked bar graphs depicting cell composition of three samples at baseline and after 24 h culture with one, two, or three slices per well. Cell populations were distinguished broadly between EpCam+ cancer cells, CD45 + immune cells, or other cells (top) and the immune compartment was further subdivided based on specific surface markers (bottom). **D** Dot plot of pre-gated viable CD45 + cells of a sample that has been incubated with/without an anti-CD3 antibody (clone SK-7) labeled with BV510 for 24 h. The slices have then been dissociated and stained with an anti-CD3 antibody (clone UCHT1) labeled with PerCP-Cy5.5, recognizing an overlapping epitope. **E** Analysis of proportion of T cells expressing the activation markers CD69, CD137, and HLA-DR in control and stimulated samples. Different wells containing either one, two, or three slices were compared

The PCTS model could be especially valuable for assessing immune-modulatory treatments depending on the presence of various immune cells and their ability to respond to stimuli. We established a 22-plex flow cytometry antibody panel covering a variety of cell surface markers for deep characterization of the PCTS model after culture (Fig. 3A). In addition, activation markers were measured on CD3+ T cells to enable an evaluation of immune response to stimuli (Fig. 3B).

The antibody panel was applied to characterize and compare the tumor composition in three samples at baseline, and after 24 h in culture with either one, two, or three PCTS per well (Fig. 3C). The cell populations were distinguished as cancer, immune, and other cells, and the immune compartment was further detailed, revealing a distinct immune infiltration of each tested patient sample. While in samples P12 and P13 the cultured PCTS differed in the proportion of immune cells compared to their corresponding baseline (Fig. 3C), sample P11 presented a stable distribution of the major cell populations over time, even across number of slices per well. The decrease of CD45 + cells in P12 and P13 was mostly reflected in the decrease of B cells and regulatory T cells (Tregs). Thus, in order to capture also populations with low abundance, especially within the immune compartment, at least three PCTS per well should be cultured.

Next, we aimed to test whether the identified immune cells were not only present but functional in culture. We first confirmed that an antibody binding to CD3 could fully penetrate the PCTS and bind to all available CD3 epitopes within 24 h. This was assessed by flow cytometry and the lack of signal of another fluorochrome-labelled CD3-binding antibody which was added post culture (Fig. 3D). The PCTS were subsequently stimulated with anti-CD3/CD28 antibodies for 24 h and surface activation marker expression was analyzed on T cells again using one, two, or three slices per well (Fig. 3E). In sample P12, the single-slice culture could not be analyzed as too few cells were captured, thus confirming the previous findings of a minimum number of slices per well. Upon stimulation, T cells expressing the early activation marker CD69 were elevated in samples P11 and P13, while CD137 + T cells were robustly increased in all three tested samples when using two or three slices per well. In contrast, the proportion of HLA-DR + T cells was inconsistent in two out of the three tested samples using this stimulation, suggesting that it might not serve as a feasible activation marker in this setting.

Taken together, we show that by using three randomly selected slices per well we can map the tissue cell composition consistently and capture even small immune populations. We further confirm that T cells in the PCTS remain functional and elevate their expression of activation markers on their surface upon TCR stimulation.

### The PCTS secretome is a robust read-out measure and reveals functional immune signaling

**Fig. 4:**
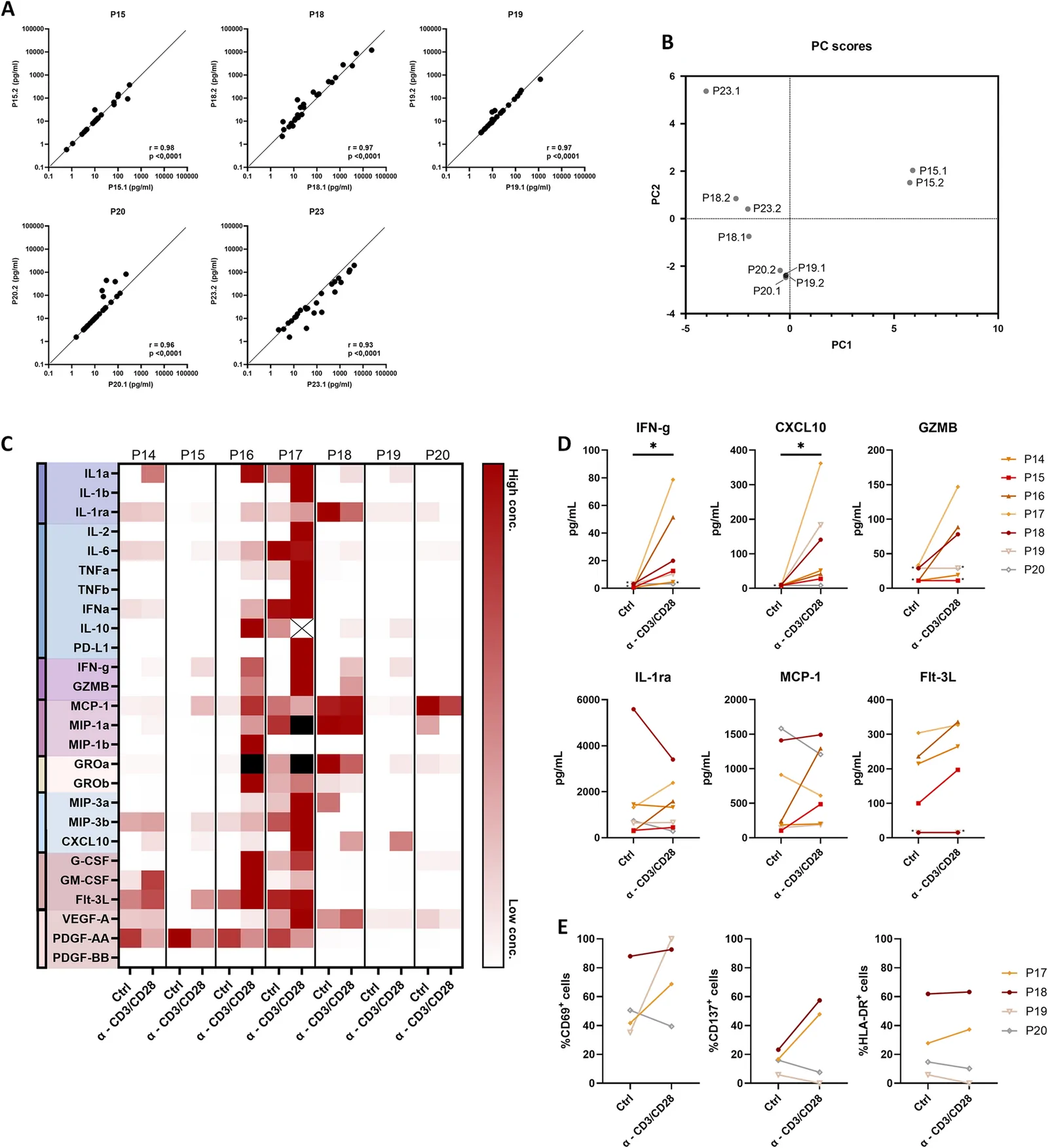
CD3/CD28 stimulation response can be captured in the secretome. **A** Secreted cytokines and chemokines of two different control wells from five patient samples were measured and plotted against each other. Diagonal lines depict full alignment. Correlation between paired control wells was assessed using Spearman rank correlation, and the Spearman correlation coefficient (r) and corresponding p value are shown. **B** Score plot showing the different tested samples based on principal component analysis. **C** Heatmap illustrating the normalized values of a 26-plex immunoassay. Cytokines and chemokines in the culture supernatant were measured of seven different PCTS samples treated with an isotype control antibody or stimulated with anti-CD3/CD28 antibodies. White represents samples with concentrations below detection range, black is above detection range and crossed analyte values were removed due to high %CV. **D** Line graphs showing selected data from the immunoassay analysis. Values below the detection range are marked with an asterisk. **E** Proportion of CD69+, CD137+, and HLA-DR + T cells in four PCTS samples after CD3/CD28 stimulation compared to control

To further evaluate the robustness of our experimental design and additional read-out options, we collected culture supernatant from two independent control wells of the same tumor sample. Cytokine and chemokine levels from a total of five different patient samples were measured using a 26-plex immunoassay and the analyte concentrations of paired control sample wells were plotted to evaluate alignment (Fig. 4A). Overall, the concentrations of the tested analytes were concordant in the duplicates, confirming that our protocol allows for a stable assessment of the secretome. In addition, principal component analysis of all samples showed grouping of the duplicates and minimal variance (Fig. 4B).

We then analyzed the culture supernatant of seven PCTS cultures upon anti-CD3/CD28 treatment, as compared to the control (Fig. 4C and D). The changes in cytokine and chemokine secretion indicated a broad immune response. In this sample cohort, the level of IFNγ was significantly higher in the treated as compared to untreated condition, although levels varied as expected due to patient sample heterogeneity. In only one out of the seven samples (P20), IFNγ was not detected following anti-CD3/CD28 stimulation. Taken together, the increased level of IFNγ in the cohort supports that the PCTS cultures preserve T cell functionality. Of note, the chemokine C-X-C motif chemokine 10 (CXCL10), which is regulated by IFNγ [21, 22], was also significantly elevated in the treated versus untreated setting, suggesting that immune related feedback signaling is functioning in the PCTS. In support of this, the only sample not showing an increased CXCL10 level upon treatment was P20, where IFNγ was not detected. Other immune response mediators like Granzyme B (GZMB) and FMS-related tyrosine kinase 3 ligand (Flt-3 L) showed a similar trend of elevation upon stimulation, though not statistically significant, while the interleukin 1 receptor antagonist (IL-1ra) and monocyte chemoattractant protein-1 (MCP-1) changed in a patient sample-specific manner.

Four of the samples investigated were additionally processed for flow cytometry and stained for T cell activation markers (Fig. 4E). Again, in all samples but P20, we detected an increased proportion of CD69 + T cells, and in P17 and P18 also of CD137 + T cells. In line with our previous findings, the percentage of HLA-DR + T cells did not change after stimulation. The fact that P20 showed an unaltered profile according to both readouts supports the reliability of our analysis and suggests that the T cells in this tumor were unresponsive to activation. These findings underline that the PCTS model allows for detailed analyses of the immune compartment and that it captures immune responses with readouts from different analysis methods with congruent results.

### PCTS reflect heterogeneous response to immune checkpoint inhibitor treatment

**Fig. 5:**
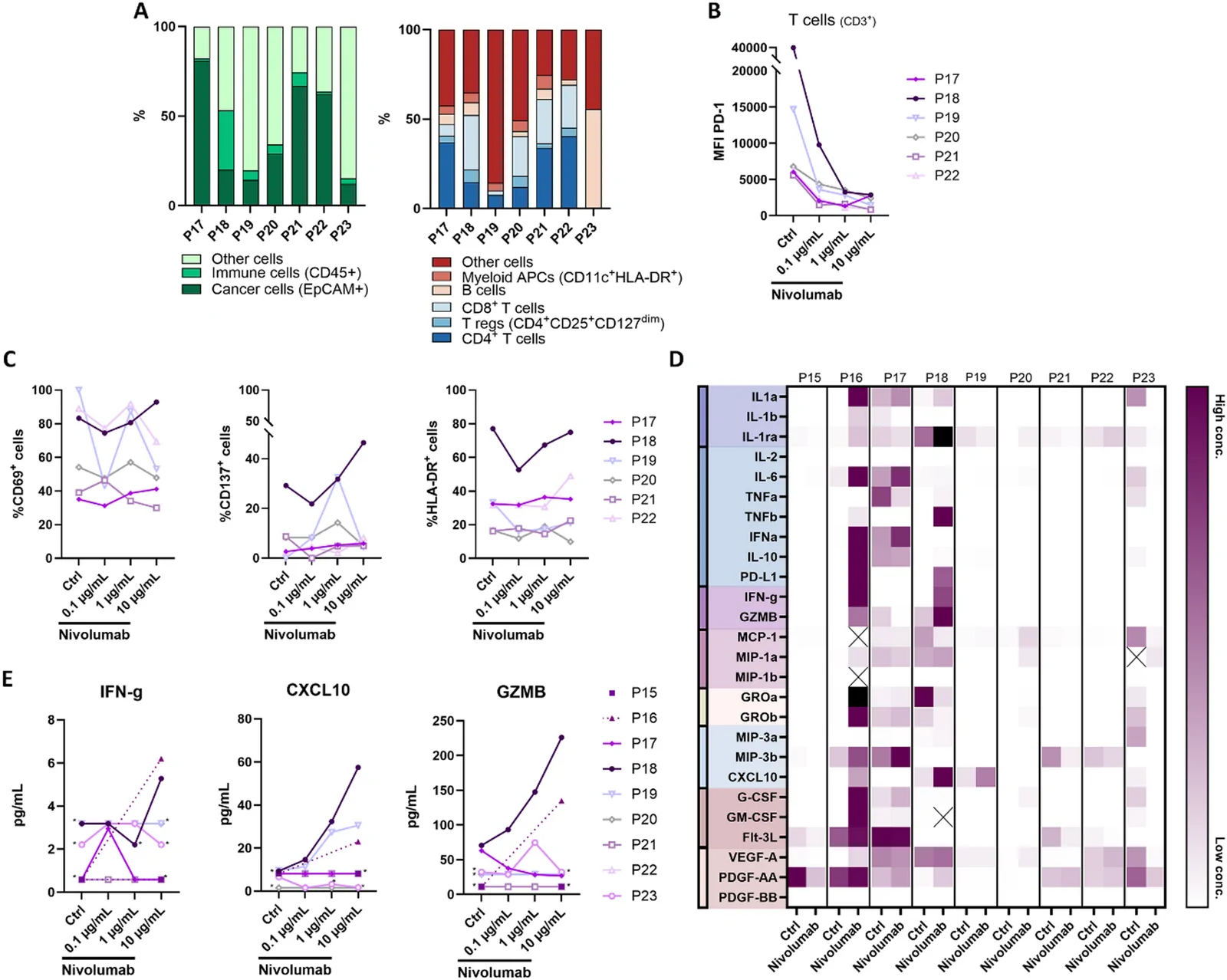
Functional analysis of anti-PD-1 treatment response in PCTS. **A** Tumor tissue composition after treatment with isotype control antibody analyzed by flow cytometry. Cell populations are distinguished as EpCam+ cancer cells, CD45 + immune cells (left) and the immune compartment is further subdivided based on surface markers (right). **B** PD-1 signal decreased with higher nivolumab dosage as the epitope for the staining antibody was occupied by the therapeutic antibody. **C** CD69+, CD137+, and HLA-DR+ cells out of all T cells plotted as line graphs for each analyzed patient sample. **D** Mediators in PCTS culture supernatant were measured using a high-plex immunoassay. Normalized concentrations from nivolumab treated (10 µg/ml) and isotype control wells shown. White represents samples with concentrations below detection range, black was above detection range and crossed analyte values were removed due to high %CV. **E** Line graphs showing selected analytes from the immunoassay analysis at different concentrations when available. Samples P15 and P16 were only treated with the highest concentration, depicted with a straight dotted line. Values below or above detection range are marked with an asterisk

Considering that reported response rates to ICIs in clinical studies are 20% [9, 10], we aimed to test whether inter-patient heterogeneity in relation to immune activation could be detected in the PCTS model. Thus, PCTS from nine patients were treated with single-dose (10 µg/ml, P15 and P16) or increasing doses (P17-P23) of nivolumab. Phenotyping the control PCTS in six of these samples using flow cytometry showed highly heterogeneous tumor compositions, with high immune infiltration and high proportion of CD8 + T cells in P18 (Fig. 5A). We observed a dose-dependent decrease in PD-1 signal, indicating a gradual saturation of PD-1 binding sites with rising nivolumab concentrations in the cultures and supporting complete tissue penetration by nivolumab (Fig. 5B). Unlike the strong CD3/CD28 stimulation, PD-1 blockade induced more modest and sample-dependent changes; therefore, concordant changes across multiple immune-related readouts were interpreted as evidence of ex vivo immune activation. Specifically, this included coordinated increases in secreted cytokines and chemokines (such as CXCL10, GZMB and IFNγ), relative to matched controls, further supported by an increase in T cell activation markers when material allowed. Analysis of cell surface activation markers revealed one PCTS sample (P18) with a dose-dependent increase in the proportion of CD69⁺ and CD137⁺ T cells upon nivolumab treatment (Fig. 5C). This sample also showed immune activation at the level of the secretome, with dose-dependent increases in CXCL10 and GZMB, as well as a modest increase in IFNγ at the highest concentration (Fig. 5D and E). In addition, sample P16 exhibited a similar coordinated cyto- and chemokine secretion pattern following PD-1 blockade. Although flow-cytometric analysis was not performed for this sample due to limited material, the secretome profile suggests immune activation in this culture.

Overall, two out of nine PCTS cultures (22%) displayed a concordant ex vivo immune activation signature following PD-1 blockade under the applied culture conditions. It should be noted that the readouts represent pharmacodynamic responses within the culture system and cannot be correlated with patient-level clinical response in the current cohort, as most patients did not receive PD-1 blockade and as extensive time-lines are required for reliable follow-up. Still, the positive ex vivo immune activation signature rate aligns with clinical observations [9, 10], thus warranting further evaluation of PCTS cultures in future clinical trials where treatments can be directly mirrored and responses compared, to determine the model’s predictive value.

Taken together, the results support that our model is applicable across heterogeneous patient populations and that T cells in the model display functional responsiveness to both very potent CD3/CD28 triggering and to clinically relevant immune checkpoint inhibition, highlighting its potential for advancing immunomodulatory drug development.

### Chemotherapy response assessment in PCTS

**Fig. 6:**
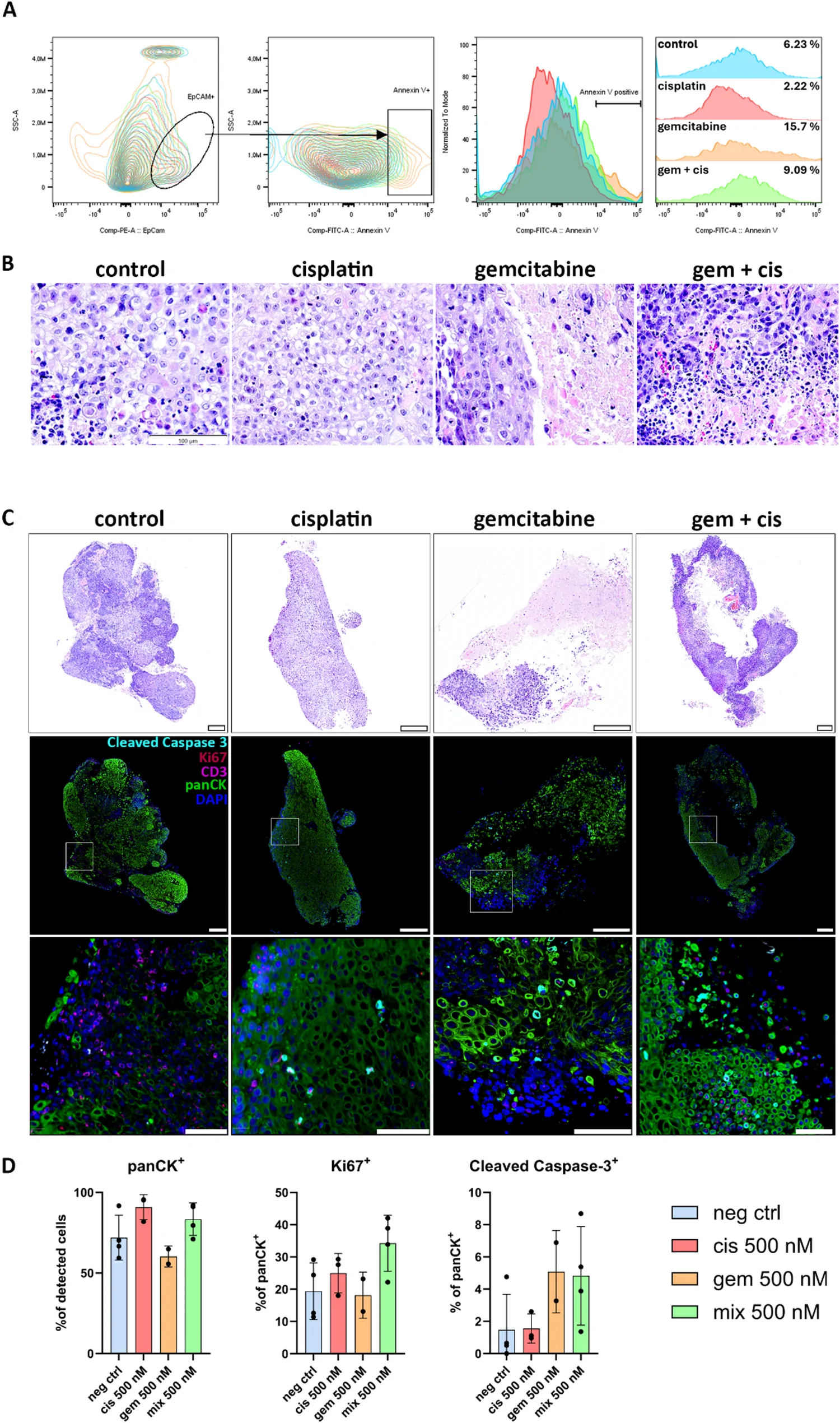
Chemotherapy induced apoptosis is captured on single-cell level and spatially. **A** PCTS from sample P18 were treated with vehicle control, cisplatin, gemcitabine or a combination (gem + cis) and apoptosis of EpCam+ cancer cells was measured by flow cytometry. **B** H&E images used for pathological evaluation of PCTS treated with cisplatin, gemcitabine, or gem + cis. Scale bar corresponds to 100 μm. **C** First row shows overview H&E images of PCTS after indicated treatment, while the second row shows the corresponding mIF images with panCK in green, CD3 in purple, cleaved caspase-3 in cyan, Ki67 in red, and cell nuclei in blue (DAPI). Scale bars correspond to 200 μm. Close-ups are presented in the third row. Scale bar corresponds to 50 μm. **D** Immunofluorescence signals were quantified and are presented as proportion of all detected cells (left - panCK) or of panCK+ cells (middle - Ki67 and right - cleaved caspase-3). Data points with standard deviation correspond to different available slices of the same treatment well (*n* = 2–4)

Platinum-based chemotherapy has long been a central treatment approach in advanced urothelial cancer [23]. To explore whether the cancer cells in our model are sensitive to chemotherapy, and that effects on cell viability can be monitored post culture, PCTS from one patient were exposed to these drugs for 24 h as a proof-of-concept. The samples were analyzed using both flow cytometry of dissociated tissue and spatial assessment of formalin-fixed slices. Apoptosis of EpCam+ cancer cells was measured by annexin V staining and in this sample, we observed sensitivity to gemcitabine and gem-cis treatment (16% and 9.1% positive cells, respectively) (Fig. 6A). H&E stainings of control and cisplatin-treated PCTS presented well-preserved tissue architecture, minimal necrotic change and intact cytological detail as assessed by pathology (Fig. 6B). In contrast, gemcitabine and gem-cis combination treated PCTS exhibited cell swelling, loss of nuclear details, cytoplasmic coagulation, prominent shadow cells and regions with cellular debris reflecting necrotic changes. Notably, gemcitabine-treated PCTS presented more prominent areas of necrosis compared to the other treatments (Fig. 6B). Additional sections of the formalin-fixed PCTS were further examined by multiplex immunofluorescence (mIF) using a 5-plex panel to stain for proliferating (panCK+Ki67+) or apoptotic (panCK+cleaved caspase-3+) cancer cells as well as T cells (CD3+) (Fig. 6C). The quantified signals revealed cancer cell apoptosis signals in line with the flow cytometry results (Fig. 6D). While the cancer cells were generally highly proliferative across all treatments, the highest proportion of apoptotic cells was detected in the gemcitabine and gem-cis combination treated sample. To further validate that mIF is relevant for monitoring effects of chemotherapy on cancer cell viability, we performed mIF staining for apoptosis in cancer cells on an additional sample (P20) following chemotherapy treatment. This supplementary analysis confirmed the detection of apoptotic cancer cells in PCTS after chemotherapy, with the highest proportion upon cisplatin treatment (Additional file 1: Figure S5), supporting the technical robustness of the mIF approach in this setting. Although the small sample size precludes any biological interpretation, our results support the feasibility of detecting chemotherapy-induced changes in viability using different readouts and that our PCTS model allows assessment of both immune and cancer cell responsiveness to various treatment approaches, potentially also to combination therapies.

## Discussion

Reliable preclinical models are valuable for developing and improving cancer treatments; however, there is still a large translational gap between preclinical research, applicability, and clinical implementation [24]. A challenge for preclinical models is the complexity of the tumor microenvironment which needs to be accurately mirrored as it influences treatment response [25]. In this study, we establish a PCTS model based on bladder tumors that captures the complexity of the TME, allows for several different readouts and can be used to outline responses to various treatment stimuli.

We tested different culture conditions and showed stable cell viability of both the cancer cells and the immune compartment during culture. In line with reports from other studies [17, 26, 27], our approach preserved the morphology of the tumor, and we defined an optimal culture medium composition. During 24 h stimulation experiments, cell viability was preserved, although culture periods could potentially be prolonged for samples with high initial viability [26, 28].

The functional integrity of immune cells within our PCTS platform was validated through systematic T cell stimulation experiments. Our deep-phenotyping analysis using flow-cytometry enabled us to decipher the sample specific cell composition and measure T cell activation based on surface marker expression. The diversity in immune cell composition across patient samples reflects the well-documented heterogeneity of bladder cancer immune infiltration [29, 30]. We captured even small immune cell populations when using three PCTS per culture well and confirmed a robust activation of T cells. The high correlation of cytokine profiles between replicate culture wells from identical patient samples further demonstrates that our protocol successfully addresses intra-tumor heterogeneity, a major confounding factor in cancer research. Consequently, by utilizing multiple randomly selected tissue slices per condition, representative sampling of the heterogeneous tumor landscape was accomplished while maintaining experimental reproducibility. Such an approach also provides sufficient statistical power to detect treatment effects while accounting for the inherent spatial variability within tumors [31, 32].

The robust elevation in the proportion of CD69 + and CD137 + T cells, coupled with the increased secretion of inflammatory mediators including IFNγ and GZMB, demonstrated preserved immune cell responsiveness to stimulatory agents. Critically, the concomitant increase in CXCL10, which is directly regulated by IFNγ [21, 22], confirms that inter-cellular immune signaling pathways remain intact within the tissue slices. This finding is particularly significant as it demonstrates that our model identifies not only individual cell responses but also the complex network of immune cell interactions essential for therapeutic efficacy. The single non-responsive sample (P20) across both readout methods validates the reliability of our analytical approach and likely represents tumors with intrinsic immune suppressive mechanisms.

Our demonstration that immune activation can be achieved following PD-1 blockade, and that the response is heterogeneous, highlights the utility of the PCTS platform for assessing ex vivo pharmacodynamic responses. The observation that two out of nine samples (22%) showed activation upon treatment with nivolumab is within the range of clinical response rates reported for PD-1 inhibitors in bladder cancer [9, 10]; however, this numerical similarity should not be interpreted as evidence of correspondence with patient outcomes. In the present study, patients received heterogeneous standard-of-care treatments and clinical response to PD-1 blockade could thus not be assessed and systematically compared. Additionally, follow-up for recently collected samples remains limited. The activation signatures observed in samples P16 and P18, characterized in P18 by increased CD137 + T cell activation and in both samples by elevated secretion of GZMB and CXCL10, recapitulate the immune activation patterns associated with checkpoint inhibitor efficacy that were recently also confirmed in a patient-derived tumor fragment model [18, 33]. Additionally, the identification of both ex vivo responsive samples as basal-squamous molecular subtype aligns with clinical observations that this subtype demonstrates higher immune infiltration and sensitivity to immune checkpoint inhibition [12, 34, 35], supporting the biological plausibility of the observed ex vivo responses.

Using our established analysis pipeline, we were able to detect cancer cell apoptosis and tumor necrosis upon treatment with cytostatic drugs both through flow cytometry and spatial histological assessment. To further validate the technical workflow, we performed supplementary mIF analysis of a second patient sample (P20), which confirmed the detection of apoptotic cancer cells in PCTS following chemotherapy treatment. While this approach was performed as a proof-of-concept and needs further expansion, the ability to simultaneously monitor responses to immunotherapy and conventional chemotherapy within the same platform addresses an unmet need in cancer treatment development. Combination therapy with nivolumab and gemcitabine-cisplatin is the current first-line treatment recommendation for patients who are ineligible to enfortumab vedotin combined with pembrolizumab [36, 37], where multi-modal treatment approaches generate better efficiency than monotherapies [38].

Several limitations of our approach warrant consideration. The relatively short culture duration (24–48 h) may not capture long-term treatment effects or adaptive resistance mechanisms that develop over extended treatment periods. Also, immune cell infiltration from circulating cells was not mirrored in the current setting, although the model allows for such further developments [39]. Further on, the dependence on surgical specimen quality introduces potential selection bias, as samples with poor viability might not be effectively cultured, and all samples in the current series therefore were obtained from an exophytic non-necrotic tumor fraction. Future studies should investigate approaches to extend culture duration and improve success rates across varying tissue quality conditions. Additionally, several experiments in this study are limited by small sample numbers, precluding statistically powered comparisons; these analyses are therefore intended to demonstrate feasibility and biological heterogeneity rather than to establish definitive response metrics. While our sample size provides proof-of-concept validation, larger cohorts will be necessary to establish definitive predictive biomarkers and validate clinical correlations across diverse patient populations. Thus, the current study does not determine alignment with clinical response, although the platform reflects ICI response rates that merit further investigation. Expanding the patient cohort in future studies will also enable investigations across the spectrum of bladder cancer molecular subtypes. The heterogeneity captured in our current cohort demonstrates that the PCTS platform faithfully recapitulates patient diversity. Larger cohorts would enable stratification analyses to identify which tumor characteristics predict ex vivo responsiveness, potentially uncovering new therapeutic vulnerabilities. Furthermore, prospective studies could incorporate emerging standard-of-care agents such as antibody-drug conjugates (e.g. enfortumab vedotin) and novel combination regimens, positioning the PCTS platform as a dynamic tool that evolves alongside the therapeutic landscape. Lastly, expansion to additional cancer types will be essential to establish the broader applicability of this platform, a topic of current investigation in our lab.

Our established PCTS model gains additional relevance in light of recent regulatory shifts toward reducing animal testing requirements for drug approvals. With the FDA Modernization Act 2.0 signed into law in December 2022, drug developers are no longer required to use animal testing before human trials if appropriate alternative methods exist [40, 41]. This landmark legislation formally recognizes the validity of human-relevant testing methods and highlights the need for platforms like our PCTS model. By providing a human-derived platform that maintains native immune cell functionality and spatial organization - critical factors for evaluating immunotherapeutic agents such as monoclonal antibodies targeting PD-1/PD-L1 - our approach addresses the growing need for physiologically relevant alternatives to animal testing.

In conclusion, the established PCTS model offers a translational platform for evaluating complex responses to biologics like checkpoint inhibitors, potentially accelerating development pipelines while aligning with evolving regulatory frameworks that emphasize human-relevant testing methods.

## Conclusions

In summary, our study demonstrates that the PCTS model derived from bladder tumors faithfully recapitulates the complexity and heterogeneity of the tumor microenvironment, supporting robust cancer and immune cell viability while enabling the detection of functional immune responses and treatment effects. The model’s ability to capture heterogeneous ex vivo immune activation upon treatment with a clinically relevant PD-1–blocking antibody, and to monitor effects of conventional chemotherapies, underscores its translational potential. Importantly, PCTS presents a human-relevant, physiologically accurate alternative to animal testing, aligning with updated regulatory frameworks that encourage non-animal research methods. Although the approach faces certain limitations, such as short culture duration and dependence on tissue quality, these are addressable through future methodological refinements.

Thus, the PCTS platform stands as a promising tool for drug development, supporting rapid and reliable assessment of novel cancer therapies in clinically relevant settings. Prospective, appropriately powered studies with standardized clinical endpoints are warranted to determine whether ex vivo readouts correlate with patient outcomes, thereby supporting its further development and validation as a predictive model.

## Supplementary Information

Below is the link to the electronic supplementary material.


Supplementary Material 1: Additional file 1: Table S1-S5 and Figure S1-S5


## Data Availability

The datasets used and/or analyzed during the current study are available from the corresponding author on reasonable request.

## Abbreviations

%CV: Percent Coefficient of Variation
APC/APCs: Antigen-Presenting Cell(s)
BaSq: Basal/Squamous (molecular subtype)
BSA: Bovine Serum Albumin
CD: Cluster of Differentiation (e.g., CD3, CD4, CD8, etc.)
Cis: Carcinoma in situ
CXCL10: C-X-C Motif Chemokine Ligand 10
DAPI: 4′,6-Diamidino-2-Phenylindole (nuclear stain)
DMEM: Dulbecco’s Modified Eagle Medium
DMSO: Dimethyl Sulfoxide
DPBS/D-PBS: Dulbecco’s Phosphate-Buffered Saline
EDTA: Ethylenediaminetetraacetic Acid
EpCAM: Epithelial Cell Adhesion Molecule
FAP: Fibroblast Activation Protein
FBS: Fetal Bovine Serum
FFPE: Formalin-Fixed Paraffin-Embedded
Flt3L: FMS-Related Tyrosine Kinase 3 Ligand
FDA: Food and Drug Administration
GEM: Gemcitabine (chemotherapy agent)
GM-CSF: Granulocyte-Macrophage Colony-Stimulating Factor
GROα/β: Growth-Regulated Oncogene alpha/beta
GU: Genomically Unstable (molecular subtype)
GZMB: Granzyme B
H\&E: Hematoxylin and Eosin (staining)
HLA-DR: Human Leukocyte Antigen – DR isotype
ICI/ICIs: Immune Checkpoint Inhibitor(s)
IHC: Immunohistochemistry
IFNγ: Interferon Gamma
IL-1α/β/ra/2/6/10: Interleukin 1 alpha/beta/receptor antagonist/2/6/10
MIBC: Muscle-Invasive Bladder Cancer
MCP-1: Monocyte Chemoattractant Protein-1
mIF: Multiplex Immunofluorescence
mAb: Monoclonal Antibody
MIP-1α/β/3α/3β: Macrophage Inflammatory Protein 1 alpha/beta/3 alpha/3 beta
NAMs: New Approach Methodologies
NMIBC: Non-Muscle-Invasive Bladder Cancer
n/a: Not Available
PD-1: Programmed Cell Death Protein 1
PD-L1: Programmed Death-Ligand 1
PDGF-AA/BB: Platelet-Derived Growth Factor AA/BB
PCTS: Precision-Cut Tumor Slices
RPMI: Roswell Park Memorial Institute (medium)
TCR: T Cell Receptor
TME: Tumor Microenvironment
TNF-α/β: Tumor Necrosis Factor Alpha/Beta
UroA/UroB: Urothelial-like A/B (molecular subtypes)
VEGF-A: Vascular Endothelial Growth Factor A
