# Supplementary material for: Bladder cancer precision-cut tumor slices (PCTS) preserve functional immune microenvironment enabling ex vivo drug evaluation
Source: BMC Med. 2026 Aug 29;24:468. doi: 10.1186/s12916-026-05173-4 (PMC13531792; doi:10.1186/s12916-026-05173-4)
Supplement: Supplementary file 1 [file 12916_2026_5173_MOESM1_ESM.docx]

Title

Bladder Cancer Precision-Cut Tumor Slices (PCTS) Preserve Functional Immune Microenvironment Enabling *Ex Vivo* Drug Evaluation

**Authors**

Sarah Richtmann^1,2,3^, Maja Radak^1,3^, Viktor Sincic^1,2,3^, Jenny Hugoson^1^, Nadja Werner^1^, Valeriia Skoryk^4^ , Darcy Wagner^3,4,5^, Fredrik Liedberg^3,6,7^, Kristina Lundberg^1,2,3,*^

**Affiliations**

1 Department of Immunotechnology, Lund University, Lund, Sweden.

2 CREATE Health Cancer Center, Lund University, Lund, Sweden.

3 LUCC Lund University Cancer Center, Lund University, Lund, Sweden.

4 Department of Experimental Medical Sciences, Lund University, Lund, Sweden.

5 Department of Biomedical Engineering, McGill University, Montreal, Canada.

6 Department of Translational Medicine, Lund University, Malmö, Sweden.

7 Department of Urology, Skåne University Hospital, Malmö, Sweden.

*corresponding author

Additional file 1: Table S1-S5 and Figure S1-S5

Table S 1: 12-plex panel used for staining of dissociated slices cultured in different media, acquired on BD FACSAria II or Cytek Aurora.

| **Staining agent** | **Fluorophore** | **Catalogue nr.** | **Company** | **Clone** | **Dilution** |
| --- | --- | --- | --- | --- | --- |
| **Viability Dye** | FV620 | 5649996 | BD bioscience | N/A | 1:1000 |
| **CD20** | PerCP-Cy5.5 | 560736 | BD bioscience | 2H7 | 1:20 |
| **HLA-DR** | APC-H7 | 561358 | BD bioscience | G46-6 | 1:20 |
| **EpCAM** | PE | 347198 | BD bioscience | EBA-1 | 1:20 |
| **CD8a** | BV785 | 301046 | Biolegend | RPA-T8 | 1:20 |
| **CD11c** | BV421 | 562561 | BD bioscience | B-ly6 | 1:20 |
| **CD45** | FITC | 560976 | BD bioscience | HI30 | 1:20 |
| **CD1c** | APC | 17-0015-42 | bioscience | L161 | 1:20 |
| **CD16** | PE-Cy7 | 557744 | BD bioscience | 3G8 | 1:20 |
| **CD3** | BV510 | 344828 | Biolegend | SK7 | 1:20 |
| **CD14** | BV605 | 301834 | Biolegend | M5E2 | 1:20 |
| **CD4** | AF700 | 557922 | BD bioscience | RPA-T4 | 1:20 |

Table S 2: 22-plex panel used for staining of dissociated bladder slices treated with immune activation stimulants or therapeutic agents, acquired on Cytek Aurora.

| **Staining agent** | **Fluorophore** | **Catalogue nr.** | **Company** | **Clone** | **Dilution** |
| --- | --- | --- | --- | --- | --- |
| **Viability Dye** | ViaDye Red | R7-60008 | Cytek | N/A | 1:100,000 |
| **EpCAM** | PE | 347198 | BD Biosciences | EBA-1 | 1:200 |
| **CD3** | BV786 | 563800 | BD Biosciences | SK7 | 1:100 |
| **CD127** | BV421 | 562436 | BD Biosciences | HIL-7R-M21 | 1:50 |
| **CD19** | PE-Cf594 | 562321 | BD Biosciences | HIB19 | 1:200 |
| **PD-1** | PE-Cy7 | 329917 | Biolegend | EH12.2H7 | 1:200 |
| **Annexin V** | FITC | A13199 | ThermoFisher | N/A | 1:500 |
| **Nectin-4** | AlexaFluor647 | FAB2659R | R&D System | # 337516 | 1:20 |
| **CD40** | RB780 | 755840 | BD Biosciences | HB14 | 1:200 |
| **CD86** | BV650 | 563411 | BD Biosciences | 2331 FUN-1 | 1:100 |
| **CD62L** | PerCP Fire 806 | 304868 | Biolegend | DREG-56 | 1:200 |
| **CD69** | BV711 | 563836 | BD Biosciences | FN50 | 1:200 |
| **CD137** | PE-Cy5 | 309808 | Biolegend | 4B4-1 | 1:200 |
| **CD8** | BV605 | 564115 | BD Biosciences | SK1 | 1:200 |
| **CD16 FcyRIII** | BV480 | 566171 | BD Biosciences | 3G8 | 1:200 |
| **CD11c** | violetFluor 450 | 75-0116-T100 | Cytek | 3,9 | 1:20 |
| **CD45** | cFluor V547 | R7-20012 | Cytek | HI30 | 1:200 |
| **CD25** | cFluor® BYG710 | R7-20586 | Cytek | BC96 | 1:200 |
| **HLA-DR** | cFluor® R840 | R7-20294 | Cytek | L243 | 1:200 |
| **CD14** | cFluor® B548 | R7-20116 | Cytek | 63D3 | 1:200 |
| **CD4** | cFluor BYG610 | R7-20040 | Cytek | SK3 | 1:200 |
| **Fibroblast Activation Protein (FAP)** | AlexaFluor700 | FAB3715N-100UG | R&D System | # 427819 | 1:5000 |

Table S 3: Treatment with immune stimulatory agents, checkpoint inhibitor, and chemotherapy in PCTS.

| **Stimulant** | **Concentration** | **Company** | **Catalogue nr.** |
| --- | --- | --- | --- |
| **Anti-CD3** | 5 µg/mL | Biolegend | 300438 |
| **Anti-CD28** | 2 µg/mL | Biolegend | 302934 |
| **Nivolumab** | 0.1, 1 and/or 10 µg/mL | Bristol Meyer Squibb | N/A |
| **Cisplatin** | 50, 500 and/or 5000 nM | Sigma-Aldrich | 232120-50MG |
| **Gemcitabine** | 50, 500 and/or 5000 nM | Sigma-Aldrich | Y0000675 |
| **Cisplatin + gemcitabine** | 500 nM | N/A | N/A |
| **Human (hu) IgG4 isotype control** | 10 µg/mL | Biolegend | 403702 |
| **Mouse (ms) IgG isotype control** | 5 µg/mL | Assay Genie | IVMB0186 |
|  |  |  |  |

Table S 4: 5-plex antibody panel used for immunofluorescence staining of fixed bladder PCTS treated with chemotherapeutics.

| **Staining agent** | **Fluorophore** | **Catalogue No.** | **Company** | **Clone** | **Dil./Conc.** |
| --- | --- | --- | --- | --- | --- |
| **panCK** | AF488 | 53-9003-80 | eBioscience | AE1/AE3 | 1:300 |
| **Ki67** | AF594 | IC7617T-100UG | R&D Systems | 1297A | 1:300 |
| **cleaved caspase 3** | AF700 | IC835N-100UG | R&D Systems | D3E9 | 1:600 |
| **CD3** | CoraLite Plus 750 | 17617-1-AP | Proteintech | polyclonal | 1:1200 |
| **DAPI** | N/A | EN62248 | Invitrogen™ 62248 | N/A | 150 nM |

Table S 5: Four different media used for ex vivo culturing of bladder slices.

| **Media** | **Cat. Nr.** | **Company** | **Supplements** |
| --- | --- | --- | --- |
| **DMEM**  (high glucose, no glutamine, no phenol red) | 31053028 | Gibco | 5 % human serum + L-glutamine 4 mM + sodium pyruvate 1 mM + 1 % penicillin/streptomycin + 1% amphotericin B |
| **DMEM/F12**  (no phenol red) | 21041025 | Gibco | 5 % human serum + 1 % penicillin/streptomycin + 1% amphotericin B |
| **RPMI 1640 Medium**  (no phenol red) | 11835030 | Gibco | 5 % human serum+ 1 % penicillin/streptomycin + 1% amphotericin B |
| **RPMI 1640 Medium**  (with phenol red) | SH30096.FS | Cytiva | 10 % FBS + L-glutamine 2 mM + 1 % penicillin/streptomycin + 1% amphotericin B |

Figure S 1: Gating strategy for samples used for cultivation protocol establishment.

Figure S 2: Adjusted gating strategy for anti-CD3/CD28 treated slices. As the CD3-related epitope is occupied by the stimulating antibody, staining of this population was not possible. It was thus adjusted to include CD11-CD16-CD4+ and CD11c-CD16-CD8+ cells.

Figure S 3: Comparison of post-dissociation cell viability in freshly analyzed (green) versus cryopreserved (blue) single-cell suspensions. Viability was assessed by fixable viability dye staining during flow cytometry. Each point represents one sample.

Figure S 4: Preservation of cell morphology in sample P8. Focus of pleomorphic atypical cells with close to signet-ring cell morphology with a high extent of tissue preservation and minimal changes from day 3 to day 6 within DMEM/F12 media, H&E. Scale bar: 50 µm.

Figure S 5: Chemotherapy induced apoptosis is captured by mIF. A) Overview H&E images of PCTS from sample P20 after indicated treatment are shown in top row. The second row displays corresponding multiplex immunofluorescence images with panCK in green, CD3 in purple, cleaved caspase-3 in cyan, Ki67 in red, and cell nuclei in blue (DAPI). Scale bars are indicated in each image. Close-up images are presented in the third row. B) Quantified immunofluorescence signals are presented as the proportion of all detected cells or of panCK+ cells (for Ki67 and cleaved caspase-3).
